# Trends in Fluoroquinolone (Ciprofloxacin) Resistance in *Enterobacteriaceae* from Bacteremias, England and Wales, 1990–1999

**DOI:** 10.3201/eid0805.010204

**Published:** 2002-05

**Authors:** David M. Livermore, Dorothy James, Mark Reacher, Catriona Graham, Thomas Nichols, Peter Stephens, Alan P. Johnson, Robert C. George

**Affiliations:** * Central Public Health Laboratory, London, United Kingdom; † Communicable Disease Surveillance Centre, London, United Kingdom; ‡ IMS-HEALTH UK, Pinner, Middlesex, United Kingdom

**Keywords:** Antibiotic resistance, ciprofloxacin, fluoroquinolones, *Escherichia coli*

## Abstract

The Public Health Laboratory Service receives antibiotic susceptibility data for bacteria from bloodstream infections from most hospitals in England and Wales. These data were used to ascertain resistance trends to ciprofloxacin from 1990 through 1999 for the most prevalent gram-negative agents: *Escherichia coli*, *Klebsiella* spp., *Enterobacter* spp., and *Proteus* *mirabilis*. Significant increases in resistance were observed for all four species groups. For *E. coli,* ciprofloxacin resistance rose from 0.8% in 1990 to 3.7% in 1999 and became widely scattered among reporting hospitals. The prevalence of resistance in *Klebsiella* spp. rose from 3.5% in 1990, to 9.5% in 1996 and 7.1% in 1999, while that in *Enterobacter* spp. rose from 2.1% in 1990 to 10.5% in 1996 and 10.9% in 1999. For both *Klebsiella* and *Enterobacter* spp., most resistance was localized in a few centers. Resistance was infrequent and scattered in *P. mirabilis*, but reached a prevalence of 3.3% in 1999.

Fluoroquinolone antimicrobial drugs were a major therapeutic advance of the 1980s because they have 100-fold greater activity than their parent compound, nalidixic acid (*1*). Unlike nalidixic acid, which is used only for urinary infections and occasionally shigellosis, the fluoroquinolones have a broad range of therapeutic indications and are given as prophylaxis, e.g., for neutropenic patients. In veterinary medicine fluoroquinolones are used as treatment and metaphylaxis but not as growth promoters. Early researchers thought that fluoroquinolone resistance was unlikely to evolve, largely because resistant *Escherichia coli* mutants are exceptionally difficult to select in vitro (*2*) and because plasmid-mediated quinolone resistance remained unknown even after 30 years of nalidixic acid usage. Nevertheless, mutational fluoroquinolone resistance emerged readily in staphylococci and pseudomonads, which are inherently less susceptible than *E. coli*. More recently, fluoroquinolone resistance has emerged in *E. coli* and other *Enterobacteriaceae*, contingent on multiple mutations that diminish the affinity of its topoisomerase II and IV targets in varying ways, reduce permeability, and upregulate efflux (*3*). Plasmid-mediated quinolone resistance has been reported, but it is exceptional (*4*).

We report here resistance trends to ciprofloxacin, the most widely used fluoroquinolone in the United Kingdom, in the prevalent *Enterobacteriaceae* species from bacteremias in England and Wales during the 1990s.

## Data Sources

### Data Collection

The surveillance, described previously, depends on the voluntary reporting of bloodstream isolates by hospital laboratories in England and Wales (*5*). The number of laboratories reporting data has grown steadily: by 1998, 208 (91%) of the 229 establishments in England and Wales listed by the Association of Medical Microbiologists were participating. Participation by laboratories in Scotland and Northern Ireland is limited, and their data were excluded from our analysis. Most laboratories used variants of Stokes’ disc method (*5*) for susceptibility tests in the period reviewed, but a minority used breakpoint tests. Results reported as intermediate were counted as resistant. Quality control was provided by the laboratories’ participation in the National External Quality Assurance Scheme and by comparison to results for the smaller numbers of *E. coli* isolates from bloodstream infections tested at the Central Public Health Laboratory (*6*).

### Prescribing Data for Fluoroquinolones

Prescribing data for fluoroquinolones, as defined daily doses (*7*), were estimated for retail pharmacies by using IMS HEALTH’s British Pharmaceutical Index (BPI) and for hospitals by using Medicare Audit’s Hospital Pharmacy Audit (HPA). The BPI records pharmaceutical sales to retail pharmacies and dispensing doctors in the United Kingdom, Channel Islands, and the Isle of Man. Approximately 97% of wholesaler sales to retail and physician outlets and >80% of direct sales by manufacturers are recorded; other sales are estimated from a sample of approximately 600 pharmacies. The number of pharmacies represented in the BPI remained constant during the study period.

The HPA provides information on pharmaceutical consumption by National Health Service hospitals, which account for >95% of hospital care in the United Kingdom. Most hospitals participate: approximately 93% of beds are currently covered. Since 1995, HPA data have been collected monthly from the stock control systems of participating hospitals. Most data are supplied electronically, which minimizes reporting errors. Data include usage of pharmaceuticals among in- and outpatient departments and for private patients in NHS hospitals but not for private patients in designated private hospitals. Before 1995, HPA data were collected from wholesalers, manufacturers, and a panel of hospitals: approximately 90% of indirect sales to hospitals were received from wholesalers and approximately 40% of direct sales from manufacturers. The panel of hospitals covered approximately 80% of beds in 1990 and 84.5% in 1995.

### Statistical Analyses

Poisson regression was performed by using the log (total number of isolates with resistance information) as an offset to determine if the proportion of ciprofloxacin-resistant isolates was changing with any type of pattern over time. S-Plus (Mathsoft Inc., Seattle, WA) was used for calculation.

## Results

### Species Prevalence and Reporting Patterns

During the 1990s, the Public Health Laboratory Service received nearly 392,551 reports of bacteremia in England and Wales, including 132,311 that indicated *E. coli*, klebsiellae, *Enterobacter* spp., and *P. mirabilis* as the pathogens isolated. These four species groups thus accounted for 32% to 36% of all bacteremia results in each year and for 71% to 72% of those concerning gram-negative bacteria (Table 1). *E. coli* was the most frequently reported pathogen, causing 22% to 25% of all bacteremias in each year, whereas *Klebsiella*, *Proteus,* and *Enterobacter* spp. were among the 10 most frequent isolates. The number of bacteremia reports rose each year (Table 1), reflecting improved reporting rather than an increased incidence of disease. A fall in the proportion of reports with susceptibility data in 1997 reflected early problems after a switch to electronic reporting and was not exclusive to ciprofloxacin.

**Table 1:** Ciprofloxacin-resistant *Enterobacteriaceae* reported from bacteremias, England and Wales, 1990–1999

		1990	1991	1992	1993	1994	1995	1996	1997	1998	1999
*Escherichia coli*											
	Total no. reports	7,610	7,377	7,849	7,872	8,274	8,465	9,155	10,143	11,248	11,573
	No. with cipro. results	4,171	4,456	5,036	5,071	5,136	5,143	4,559	3,706	6,282	6,708
	No. reported ciproR	33	32	47	65	88	108	119	144	244	246
*Klebsiella* spp*.*											
	Total no. reports	1,544	1,634	1,710	1,725	1,791	1957	2,143	2,383	2,816	2,802
	No. with cipro. results	821	1,082	1,124	1,141	1,173	1,256	1,137	900	1,551	1,578
	No. reported ciproR	29	48	55	77	77	115	108	80	125	112
*Enterobacter* spp*.*											
	Total no. reports	895	912	1013	948	1,118	1,089	1,229	1,480	1,638	1,629
	No. with cipro. results	582	636	743	759	815	723	617	534	908	949
	No. reported ciproR	12	26	36	29	54	65	65	55	72	103
*Proteus mirabilis*											
	Total no. reports	868	898	911	925	984	942	1244	1,131	1,241	1,145
	No. with cipro. results	454	578	560	573	635	673	578	447	715	658
	No. reported ciproR	2	3	1	7	14	7	4	5	14	22
No. of other organisms		19,866	20,458	21,335	22,968	23,559	24,545	27,908	31,258	34,517	34,216
Total bacteremia reports		30,783	31,279	32,838	34,438	35,726	36,948	41,679	46,395	51,100	51,365

Cipro, ciprofloxacin; R, resistant.

### Resistance Trends for Ciprofloxacin

**Figure 1:**
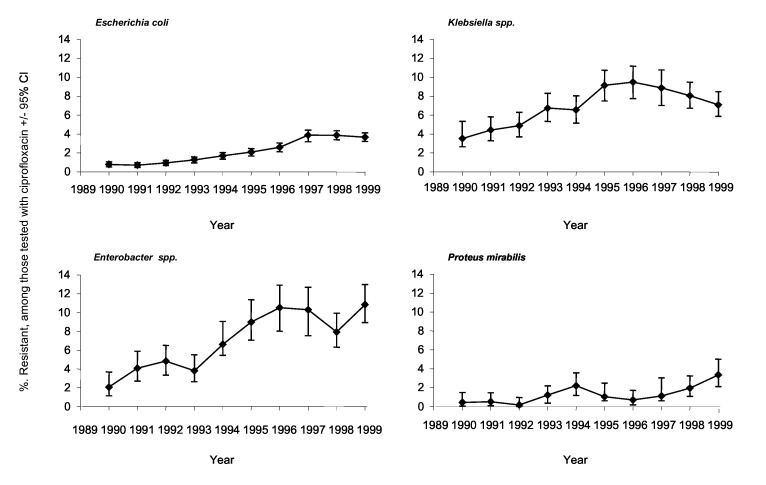
Resistance trends in *Escherichia coli,* *Klebsiella* spp., *Enterobacter* spp., and *Proteus mirabilis*, England and Wales, 1990–1999.**Bars indicate 95% confidence intervals.

Among the reports for *E. coli*, klebsiellae, *Enterobacter* spp., and *P. mirabilis,* 75,168 (56.8%) had susceptibility data for ciprofloxacin, confirming widespread testing. Ciprofloxacin resistance was extremely rare when surveillance began but subsequently increased for all four organisms (Figure 1). The proportion of *E. coli* isolates reported as resistant rose slowly but steadily, from 0.8% in 1990 to 3.7% in 1999. For *Klebsiella* spp., the resistance rate rose from 3.5% of reports in 1990 to 9.5% in 1996, before declining to 7.1% by 1999. *Enterobacter* spp. showed a similar pattern to klebsiellae: the prevalence of resistance rose from 2.1% in 1990 to 10.5% in 1996, then dipped to 7.9% in 1998 before rising to 10.9% in 1999. Only a few *P. mirabilis* isolates were reported resistant in any year before 1999. Poisson regression showed strong evidence of a trend to increasing resistance for all four organisms and suggested that these increases had a nonlinear component for *E. coli,* enterobacters, and klebsiellae. If the trends nevertheless were approximated to be linear, the average annual increases in the proportion of resistant isolates were as follows: *E. coli,* 21.54% (95% confidence intervals [CI] 18.86-24.30); *Klebsiella* spp., 6.97% (CI 4.41-9.59); *Enterobacter* spp. 13.97% (CI 10.46-17.58); and *P. mirabilis*, 21.31% (CI 11.38-32.13).

### Distribution of Resistance

To assess the distribution of resistance, we counted, for each organism in each year: 1) the number of laboratories reporting resistant isolates, 2) any laboratories contributing >10% of all reports of resistance, and 3) the proportion of reports of resistance from the top three contributors (Table 2). The last two criteria were applied only when >30 resistant isolates of a species were reported in a year, so that a hospital would not appear as a “major contributor” on the basis of three or fewer resistant isolates.

**Table 2:** Distribution of reports of ciprofloxacin resistance for *Enterobacteriaceae* from bacteremia in hospitals, England and Wales, 1990–1999

		1990	1991	1992	1993	1994	1995	1996	1997	1998	1999
*Escherichia coli*											
	No. labs reporting ciproR isolates	25	29	39	40	57	52	58	68	94	89
	Labs contributing >10% of ciproR total (n)^a^	1	0	0	0	0	0	0	0	0	0
	% of all ciproR reports from top three contributors^a^	33	19	17	20	13	19	26	17	11	12
*Klebsiella* spp*.*											
	No. labs reporting ciproR isolates	23	48	38	42	42	47	42	36	57	50
	Labs contributing >10% of ciproR total (n)^a^	-	0	1	0	0	1	2	2	1	0
	% of all ciproR reports from top three contributors^a^	-	17	29	15	21	32	35	39	23	21
*Enterobacter* spp.											
	No. labs reporting ciproR isolates	10	19	36	27	37	30	35	33	39	58
	Labs contributing >10% of ciproR total (n)^a^	-	-	0	-	0	2	2	0	0	0
	% of all ciproR reports from top three contributors^a^	-	-	28	-	22	32	30	22	22	16
*Proteus mirabilis*											
	No. labs reporting ciproR isolates	2	2	1	6	12	6	3	6	12	20
	Labs contributing >10% of ciproR total (n) ^a^	-	-	-	-	-	-	-	-	-	-
	% of all ciproR reports from top three contributors^a^	-	-	-	-	-	-	-	-	-	-

^a^Not calculated if <30 resistant isolates. Cipro, ciprofloxacin; R, resistant; labs, laboratories.

The number of laboratories reporting resistant *E. coli* rose from 25 in 1990 to 89 in 1999, and no single laboratory ever contributed >10% of all reports of resistance in a year for this species*.* Laboratories reporting five or more resistant *E. coli* in years before 1998 mostly served major teaching hospitals, but many district general hospitals reported five or more resistant *E. coli* isolates in 1998 and 1999. Resistance was more localized and more prevalent in *Klebsiella* and *Enterobacter* spp. than in *E. coli.* The number of laboratories reporting resistant klebsiellae fluctuated from 36 to 57 after 1992, without obvious trend. During a peak in resistance prevalence, from 1995 to 1997, one or two laboratories each contributed >10% of all reports of resistant klebsiellae, and the top three contributors accounted for 32% to 39% of reports of resistance. For *Enterobacter* spp., laboratories reporting resistance increased from 10 in 1990 to 36 in 1992, then fluctuated with little trend until 1997, before rising to 40 in 1998 and 58 in 1999. In the peak of resistance in 1995 and 1996, two laboratories each accounted for >10% of all reports of resistant enterobacters, and 30% to 32% of reports of resistance came from the top three contributors. Resistance was uncommon in *P. mirabilis,* and clusters were not evident.

In a further analysis, we identified eight laboratories that frequently reported large numbers of resistant *E. coli*, *Klebsiella* spp., and *Enterobacter* spp. during the entire surveillance period. These were in major metropolitan areas and served teaching hospitals. These laboratories accounted for 7.7%, 11.2%, and 10.3% of reports with ciprofloxacin data for *E. coli*, *Klebsiell*a, and *Enterobacter* spp. respectively, but for 18.2%, 30.9%, and 22.4%, respectively, of reports of resistance in these organisms, confirming a major excess of resistance.

The prevalence of ciprofloxacin resistance was examined in relation to patients’ ages for *E. coli*, since those aged <14 years should not receive fluoroquinolones. Taking the period 1995 through 1999 as a whole, 12 (3.9%) of 305 *E. coli* with data from patients 1 to 14 years old were reported as ciprofloxacin resistant, compared with 778 (3.2%) of 24,302 *E. coli* isolates from patients aged >15 years. These data indicated a relative risk of 1.22 (95% CI 0.7-2.1) for the younger patients. Similar calculations were not performed for other species because of the small numbers of source patients aged 1-14 years.

### Use of Fluoroquinolones

**Figure 2:**
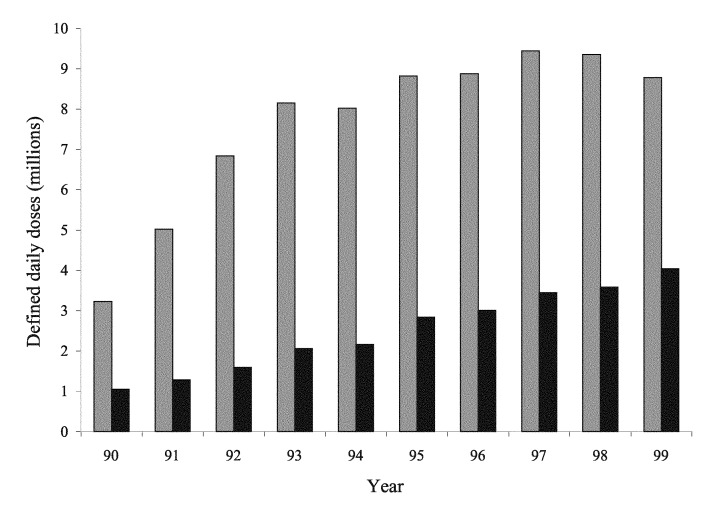
Fluoroquinolones dispensed by retail (grey) and hospital (black) pharmacies, United Kingdom, 1990–1999.

Fluoroquinolone use increased in the earlier years of surveillance, nearly doubling from 1990 to 1993. However, usage has been relatively stable from 1997 onwards, with community use declining slightly (Figure 2). Although most use is still in the community, hospital use has grown steadily in absolute terms and as a proportion, constituting 31.5% of total use in 1999 compared with 18.9% in 1992. Ciprofloxacin was the dominant fluoroquinolone throughout the period (not shown).

## Conclusion

When this surveillance began in 1990, the ciprofloxacin resistance rates in *E. coli* and *P. mirabilis* were <1%, and rates for enterobacters and klebsiellae were 2.1% and 3.5%, respectively. The prevalence of resistance in *E. coli* subsequently rose slowly and progressively to reach 3.7% in 1999; this resistance was widely scattered in hospitals. Resistance also increased significantly (p<0.01, chi-square test for trend) in enterobacters and klebsiellas. The prevalence rates for these two genera were strongly influenced by clusters of resistant isolates reported by a few laboratories. Thus, the prevalence of ciprofloxacin resistance in klebsiellae peaked at 9.5% in 1996, when three laboratories accounted for 35% of reports of resistance. A subsequent decline was associated with the absence of clusters but not with a decline in the number of hospitals that reported resistance. For enterobacters, the proportion of resistant isolates rose from 1990 to 1996, but the number of laboratories reporting resistance was relatively constant from 1992 to 1997. Peak rates of resistance in 1995 and 1996 were in a period when the top three contributors accounted for 30% to 32% of reports. Resistance in *P. mirabilis* was infrequent and scattered but rose significantly (p<0.01) in prevalence.

Although our analysis of resistance prevalence depended on the compilation of susceptibility results obtained at different sites by different methodologic variants, there is no suggestion that definitions of resistance to ciprofloxacin have become more conservative in the United Kingdom. Moreover, a rising prevalence of ciprofloxacin resistance is evident in the smaller numbers of *E. coli* isolates tested by a standardized method at the Central Public Health Laboratory, supporting the trends found here (*6*,*8*).

Several factors may explain the greater prevalence and clustering of resistance in enterobacters and klebsiellae. Most importantly, *Enterobacter* and *Klebsiella* spp. are primarily hospital pathogens, whereas *E. coli* bacteremias are more often community acquired. Thus, *E. coli* accounted for 22.8% of all bacteremias in this surveillance, which included both hospital- and community-acquired infections, but only 12.5% of hospital-acquired bacteremias, as recorded by the Nosocomial Infection National Surveillance Scheme (*9*). Although most fluoroquinolone use is in the community (Figure 2), the most intensive use and therefore the greatest selection pressure relative to numbers and concentration of patients is in hospitals. Moreover *Klebsiella* and *Enterobacter* infections are more often clonal than those involving *E. coli;* single strains, perhaps resistant, spread to numerous patients *(10)*. Clonal outbreaks seem the likely explanation when small numbers of hospitals contributed substantially to resistance totals—as was often the case for *Enterobacter* and *Klebsiella* spp. (Table 2)—but cannot be proved without retained isolates. Bacteremias caused by quinolone-resistant *E. coli* may or may not be clonal, even when multiple cases occur in a unit (*11*,*12*). The laboratories reporting clusters of resistant *Enterobacter* and *Klebsiella* spp. mostly served major teaching hospitals, where fluoroquinolone prophylaxis by hematology departments has been associated with a reduced incidence of bacteremias in neutropenic patients (*13*) but with more bacteremias being caused by fluoroquinolone-resistant strains (*14*,*15*).

We did not attempt to comprehensively relate resistance and prescribing, but three general points can be made. First, the recent decline in community prescribing of fluoroquinolones (Figure 2) has not affected the upward resistance trend in *E. coli*, although most *E. coli* bacteremia is believed to involve non-nosocomial strains. Second, the rising hospital use of fluoroquinolones has not been mirrored by an acceleration in upward trend of resistance in *Klebsiella* and *Enterobacter* spp. Third, the prevalence of resistant *E. coli* from bacteremias in patients 1-14 years old was similar to or higher than that in older patients, although the younger patients should not receive fluoroquinolones. These observations imply complex relationships between use and resistance, demanding prospective investigation.

Except for *P. mirabilis,* the resistance prevalence rates found here resemble those for bacteremias in the United States, a country with much heavier fluoroquinolone use than the United Kingdom. The Surveillance Network database (http://www.mrlworld.com) shows resistance trends (with intermediate counted as resistant) in bloodstream isolates from 250 U.S. hospitals as follows: *E. coli,* 1.8% in 1996 and 4.3% in 1999; *Klebsiella* spp., 7.1% in 1996 and 6.7% in 1999; *Enterobacter* spp., 6.6% in 1996 and 6.5% in 1999; and *P. mirabilis*, 5.7% in 1996 and 12.7% in 1999. Much higher rates are reported from Barcelona, Spain, where 17% of *E. coli* isolates from community infections were ciprofloxacin resistant (*16*), and India, where up to 50% of hospital *E. coli* are reported resistant (*17*). High rates in *E. coli* may reflect contamination via the food chain: the Spanish study found quinolone-resistant *E. coli* in 90% of chicken feces and noted similar fecal carriage rates of resistant *E. coli* in children and adults. Acquisition of resistant *E. coli* via the food chain may also explain why, in our study, resistant *E. coli* were reported from age groups who should not receive fluoroquinolone therapy and its contingent selection pressure.

Ciprofloxacin remains a potent antibiotic; but the slow accumulation of resistant *Enterobacteriaceae* is disturbing, not least because resistance is a class effect, affecting all fluoroquinolones. Ultimately, this resistance may be partly overcome by inhibiting the efflux pumps that contribute to the resistance (*18*), but this strategy is still several years from fruition. In the interim, the best approach lies in the prudent use of fluoroquinolones in humans and animals, coupled with an emphasis on preventing patient-to-patient spread of resistant strains.
